# Replication of epidemiological associations of carpal tunnel syndrome in a UK population-based cohort of over 400,000 people

**DOI:** 10.1016/j.bjps.2021.11.025

**Published:** 2022-03

**Authors:** A. Wiberg, R.W. Smillie, S. Dupré, A.B. Schmid, D.L. Bennett, D. Furniss

**Affiliations:** a Nuffield Department of Orthopaedics, Rheumatology, and Musculoskeletal Science, University of Oxford, Oxford, OX3 7LD, UK; b Nuffield Department of Clinical Neurosciences, University of Oxford, John Radcliffe Hospital, Oxford, OX3 9DU, UK; c Department of Plastic and Reconstructive Surgery, Oxford University Hospitals NHS Foundation Trust, John Radcliffe Hospital, Oxford, OX3 9DU, UK

**Keywords:** Carpal tunnel syndrome, UK Biobank, Diabetes, Rheumatoid arthritis, Hypothyroidism, BMI, Reproducibility

## Abstract

**Introduction:**

Several phenotypic factors are associated in the literature with an increased risk of carpal tunnel syndrome (CTS). Along with female sex and older age, certain systemic diseases show an association with CTS, with varying degrees of evidence.

**Methods:**

This study was performed using the UK Biobank resource – a cohort study of over 500,000 participants who have allowed linkage of phenotypic data with their medical records. We calculated the prevalence of CTS and a sex-specific prevalence ratio and compared the body mass index (BMI) between cases and controls. We performed a series of nested case-control studies to compute odds ratios for the association between CTS and three systemic diseases.

**Results:**

There were 12,312 CTS cases within the curated UK Biobank dataset of 401,656 (3.1% prevalence), and the female:male ratio was 1.95:1. CTS cases had, on average, a BMI > 2.0 kg/m^2^ greater than controls. Odds ratios for the association with CTS for three systemic diseases were 2.31 (95% CI 2.17–2.46) for diabetes, 2.70 (95% CI 2.44–2.99) for rheumatoid arthritis, and 1.47 (95% CI 1.38–1.57) for hypothyroidism. Adjusted for BMI, these odds ratios fell to 1.75 (95% CI 1.65–1.86), 2.43 (95% CI 2.20–2.69), and 1.35 (95% CI 1.26–1.43), respectively.

**Discussion:**

We harnessed the size and power of UK Biobank to provide robust replication of evidence for the associations between CTS and female sex, raised BMI, and three systemic diseases, which are only mediated in part by raised BMI.

## Introduction

Carpal tunnel syndrome (CTS) is the most common entrapment neuropathy, and it is estimated that one in ten people will develop CTS at some point in their life^1^. It is the most common peripheral nerve disease in the UK^2^, and carpal tunnel decompression surgery is the most frequently performed elective hand operation in England^3^. In the USA, approximately 500,000 carpal tunnel operations are performed annually, with estimated treatment costs in excess of $2 billion per annum – a sum unadjusted for inflation since 1995^4^. CTS therefore exacts considerable societal and healthcare costs, and gaining a better understanding of the aetiology and risk factors for the disease has significant public health implications.

There is broad consensus that female sex, pregnancy, and older age are strong risk factors for CTS^2^^,^^5^. In addition, several common systemic diseases have been reported to show an association with CTS with varying degrees of evidence. The most well-established of these disease associations with CTS are obesity, diabetes, rheumatoid arthritis (RA), and hypothyroidism^5^. Other diseases, for which there is some evidence for an association with CTS, include gout/pseudogout, amyloidosis, acromegaly, osteoarthritis, and vitamin B6 deficiency^5^, ^6^, ^7^. Although the evidence for obesity, diabetes, RA and hypothyroidism as putative risk factors for CTS is reasonably robust, these associations are by no means conclusive, with several studies presenting conflicting evidence – for instance, Hendriks et al.^8^ found that the association between diabetes and CTS disappeared once adjustments were made for age, sex and body mass index (BMI). Furthermore, one of the cornerstones of science is replication – one can have greater confidence in the veracity of associations if they can be reproduced in an independent cohort^9^.

The UK Biobank prospective cohort was designed to support the investigation of risk factors for the major diseases of middle and old age, with the recruitment of over 500,000 participants aged 40–69 between 2006 and 2010^10^. The participants have undergone detailed medical assessment and have allowed linkage of these data with their medical records, lifestyle questionnaires, and physical, cognitive, and biochemical measures. The design of the study allows for the assessment of exposures to a range of health outcomes. Medical records are in the form of both routinely collected hospital data (such as ICD-10 diagnostic codes and OPCS4 operation codes), and self-reported diagnoses. The dataset is available on request to approved researchers around the world, making UK Biobank a uniquely rich resource for determining both the genetic and non-genetic determinants of many diseases^11^.

In this study, we harness the power of UK Biobank to provide replication of the phenotypic associations of CTS, including sex distribution, BMI, diabetes, RA, and hypothyroidism. We show that the richness of this dataset and the size of the cohort provide a unique and unparalleled opportunity for robust replication.

## Material and methods

### Ethical approval

UK Biobank has approval from the North West Multi-Centre Research Ethics Committee (11/NW/0382). This study was conducted under UK Biobank study ID 10948 (The Genetics of Carpal Tunnel Syndrome), and has ethical approval through UK Biobank.

### Dataset

We had previously undertaken quality control of the raw UK Biobank dataset for a related study into the genetic origins of CTS, and this has been described in detail elsewhere^12^. The final, curated dataset comprised 401,656 participants (184,499 male, 217,157 female) of white British ancestry. The sex of the participants was determined by genotypic rather than self-reported sex, and the age of the participants was calculated on 31^st^ December 2019, based on year of birth.

### Phenotyping

CTS cases were identified using a combination of diagnostic and operative codes from the participants’ electronic hospital records, and their responses to a self-completed touch-screen questionnaire about their medical history obtained at the baseline visit. Individuals were selected as CTS cases if they had any one of the diagnostic codes shown in **Supplementary Table 1**. Individuals without any of these codes were classed as controls. Phenotyping for individuals with diabetes, RA, and hypothyroidism was performed in a similar manner (**Supplementary Table 1**).

### Prevalence

CTS prevalence was calculated for the whole cohort, and for females and males separately. The latter sex-specific prevalence figures were used to ascertain the ratio of female:male CTS prevalence within UK Biobank.

### BMI analysis for CTS patients

BMI data were available for 400,386 participants. BMI was compared between CTS cases and controls for the whole cohort, and for males and females separately. Two-tailed *t*-tests were performed in R^13^ to determine the statistical significance of any observed differences.

### Nested case-control studies

A series of nested case-control studies were undertaken to confirm epidemiological associations in the UK Biobank dataset between CTS and diabetes, RA, and hypothyroidism. A nested case-control study is also known as a “case-control in a cohort” design, whereby cases and controls are drawn from the population of a fully enumerated cohort such as UK Biobank^14^. This method offers several advantages to the traditional case-control design, such as being able to select cases and controls from the same underlying population, and the ability to negate potential confounding variables through matching.

For each of the three disease phenotypes, initial matching of cases to controls was performed at a 1:5 ratio, using the R package *MatchIt*^15^. The nearest neighbour matching method was selected, and the matching variables were “sex” and “year of birth”.

Because the association between obesity and diabetes is well-known, and hypothyroidism^16^ and RA^17^ are also linked to raised BMI, it is potentially a significant confounder in these nested case-control studies. Therefore, we generated a further control cohort for each condition matched by sex, year of birth, and BMI. Thus, a total of six case-control cohorts were generated for the three disease phenotypes. The *cobalt* R package was used to assess covariate balance following the matching.

The number of CTS cases was ascertained within each of the six case-control sets, and three odds ratios were computed for each disease: (1) for the whole cohort, and sub-group analyses for (2) males only, and (3) females only. Odds ratios were calculated as a 2 × 2 contingency table, with the disease phenotype (e.g. diabetes) as the exposure and CTS status as the outcome. Two-tailed t-tests were performed in R, and 95% confidence intervals (CI) were computed. A Bonferroni correction was applied to the significance threshold to account for multiple testing across three independent disease phenotypes, i.e. *p* < 0.0167 (0.05/3).

## Results

### Baseline characteristics and CTS prevalence

The basic demographic characteristics of the CTS cases and controls are shown in Table 1. There were 12,312 CTS cases within the curated UK Biobank cohort of white British ancestry individuals of 401,656, giving an overall prevalence of 3.1%. In the sex-specific analyses, the male prevalence was 2.0% and the female prevalence was 3.9%, giving a female:male prevalence ratio of 1.95:1.

**Table 1:** **Demographics of the UK Biobank cohort.** Demographic details are given for the 401,656 individuals post-quality control, and are shown for the whole cohort and for males and females separately. Age is given in years, with standard deviations in parentheses.

		CTS Cases	Controls
Whole cohort	**N**	12,312	389,344
	**CTS Prevalence (%)**	3.1	
	**Age**	68.5 (7.2)	66.8 (8.0)
**Males**	**N**	3,738	180,761
	**CTS Prevalence (%)**	2.0	
	**Age**	69.0 (7.4)	67.0 (7.4)
**Females**	**N**	8,574	208,583
	**CTS Prevalence (%)**	3.9	
	**Age**	68.3 (7.1)	66.6 (8.0)

### Body-mass index (BMI)

BMI was elevated in CTS cases compared to controls by an average of 2.0 kg/m^2^ (95% CI 1.96–2.13), and this effect was consistent in both males and females (males: 2.1 kg/m^2^ (95% CI 1.95–2.22); females: 2.2 kg/m^2^ (95% CI 2.11–2.33)). Results are shown in Table 2.

**Table 2:** BMI in the UK Biobank cohort. BMI (plus S.D.) are given for the post-QC UK Biobank cohort of 401,656 for whom BMI values were available (400,386 participants). “All” refers to males and females combined, and breakdowns are given by sex and CTS status. Mean differences and t-tests correspond to CTS cases vs controls.

	BMI (mean (SD), kg/m^2^)				
	**CTS cases and controls**	**CTS cases**	**CTS controls**	***T*-test *P***	**Mean BMI difference (95% CI)**
**All**	27.4 (4.8)	29.4 (5.7)	27.4 (4.7)	< 0.0001	2.0 (1.96–2.13)
**N**	400,386	12,239	388,147		
**Males**	27.9 (4.2)	29.9 (4.8)	27.8 (4.2)	< 0.0001	2.1 (1.95–2.22)
**N**	183,867	3,706	180,161		
**Females**	27.0 (5.1)	29.2 (6.0)	27.0 (5.1)	< 0.0001	2.2 (2.11–2.33)
**N**	216,519	8,533	207,986		

### Diabetes, rheumatoid arthritis and hypothyroidism

Case ascertainment within the curated cohort of 401,656 identified 24,558 individuals with diabetes, 6,711 with RA, and 23,838 with hypothyroidism. The matching algorithm produced six well-matched case-control datasets, the demographics of which are summarised in Table 3. Also shown in this table are the number of CTS cases within each of these case-control datasets.

**Table 3:** Demographics of the six matched nested case-control cohorts. DM = diabetes, RA = rheumatoid arthritis. The “BMI” suffix refers to matching on body mass index as an additional matching variable in addition to year of birth and sex. The total post-QC UK Biobank cohort size from which these nested case-control sets were derived was 401,656. The absolute number of CTS cases (“N CTS”) and prevalence (“% CTS”) are shown for the three disease phenotype cohorts and their matched controls.

*CASES*	DM	DM_BMI	RA	RA_BMI	Hypothyroid	Hypothyroid_BMI
N cases	24,558	24,558	6,711	6,711	23,838	23,838
Mean BMI	31.6 (5.8)	31.6 (5.8)	28.5 (5.5)	28.5 (5.5)	28.6 (5.5)	28.6 (5.5)
Mean Age	70.2 (6.9)	70.2 (6.9)	69.6 (7.1)	69.6 (7.1)	68.9 (7.3)	68.9 (7.3)
N males	15,309	15,309	2,175	2,175	3,994	3,994
N females	9,249	9,249	4,536	4,536	19,844	19,844
% females	37.7	37.7	67.6	67.6	83.2	83.2
N CTS	1,516	1,516	586	586	1,278	1,278
% CTS	6.2	6.2	8.7	8.7	5.4	5.4
***CONTROLS***						
N controls	122,790	122,790	33,555	33,555	119,190	119,190
Mean BMI	27.3 (4.3)	30.4 (4.9)	27.3 (4.7)	28.4 (5.3)	27.1 (4.9)	28.5 (5.4)
Mean Age	70.2 (6.9)	70.4 (6.8)	69.6 (7.1)	69.7 (7.0)	68.9 (7.3)	69 (7.3)
N males	76,545	76,545	10,875	10,875	19,970	19,970
N females	46,245	46,245	22,680	22,680	99,220	99,220
% females	37.7	37.7	67.6	67.6	83.2	83.2
N CTS	3,398	4,439	1,148	1,271	4,416	4,814
% CTS	2.8	3.6	3.4	3.8	3.7	4.0

Table 4 shows the odds ratios (OR) for association with CTS for the three disease phenotypes in the six nested case-control studies. All three disease phenotypes showed a positive association with CTS − diabetes: OR 2.31 (95% CI 2.17−2.46); RA: OR 2.70 (95% CI 2.44−2.99); hypothyroidism: OR 1.47 (95% CI 1.38−1.57). The greatest odds ratio for CTS overall was seen in males with RA (OR = 3.75, 95% CI 3.04–4.63). Matching on BMI as an additional variable had the effect of attenuating the odds ratios for CTS in all three disease phenotypes, but statistically robust associations persisted − diabetes: OR 1.75 (95% CI 1.65−1.86); RA: OR 2.43 (95% CI 2.20−2.69); hypothyroidism: OR 1.35 (95% CI 1.26−1.43).

**Table 4:** **Odds ratios for having CTS diagnosis in six nested case-control cohorts.** The “BMI” suffix refers to matching on body mass index as an additional matching variable in addition to year of birth and sex.

Disease		OR of CTS (95% CI)	*Z*-statistic	*P*-value
**Diabetes**	Whole cohort	2.31 (2.17–2.46)	26.4	<0.0001
	Males	1.99 (1.81–2.19)	14.3	<0.0001
	Females	2.63 (2.42–2.86)	22.7	<0.0001
**Diabetes_BMI**	Whole cohort	1.75 (1.65–1.86)	18.4	<0.0001
	Males	1.58 (1.44–1.74)	9.8	<0.0001
	Females	1.92 (1.77–2.08)	15.9	<0.0001
**RA**	Whole cohort	2.70 (2.44–2.99)	18.9	<0.0001
	Males	3.75 (3.04–4.63)	12.3	<0.0001
	Females	2.46 (2.18–2.77)	14.8	<0.0001
**RA_BMI**	Whole cohort	2.43 (2.20–2.69)	17.1	<0.0001
	Males	2.97 (2.42–3.64)	10.5	<0.0001
	Females	2.28 (2.03–2.57)	13.7	<0.0001
**Hypothyroid**	Whole cohort	1.47 (1.38–1.57)	11.9	<0.0001
	Males	1.61 (1.32–1.96)	4.7	<0.0001
	Females	1.46 (1.36–1.56)	10.9	<0.0001
**Hypothyroid_BMI**	Whole cohort	1.35 (1.26–1.43)	9.2	<0.0001
	Males	1.42 (1.17–1.72)	3.5	<0.0001
	Females	1.34 (1.25–1.43)	8.5	<0.0001

## Discussion

The central purpose of all medical research is to identify opportunities for new treatments and prevention. The size and scale of the UK Biobank resource offers researchers a unique and unprecedented opportunity to investigate disease epidemiology, and the various analyses presented herein have taken advantage of the statistical power conferred by the size of the UK Biobank to investigate the relationships between CTS and a multitude of phenotypes.

There is wide agreement that CTS is significantly more common in women than it is in men. There is variation in the sex-specific prevalence of CTS reported in the literature, with estimates of female:male prevalence ratios from the largest studies reported as 1.4:1^18^, 2:1^19^, 2.9:1^20^, and 3.6:1^21^. The first objective of this study was to calculate a sex-specific prevalence ratio for CTS, and the female:male ratio calculated was 1.95:1, which accords with the range of previously published figures.

It is worth noting that the UK Biobank cohort is composed of individuals who were aged between 40–69 when they were recruited between 2006–10, with a current mean age of 67 (Table 1). CTS is most common in patients over 40 years of age^22^, with the incidence peaking bimodally – in the late 50s and in the late 70s^19^. The preponderance of older participants within UK Biobank means that the sex-specific prevalence and the overall prevalence of CTS in this cohort (3.1%) is not a reflection of the true population prevalence. However, this potentially inflated CTS prevalence is balanced by the fact that UK Biobank's secondary care diagnostic codes (ICD-10 and OPCS4) derived from Hospital Episode Statistics for England (HES) are only available from 1996 onwards^23^, meaning that disease prevalence estimates based on these codes are likely to be lower than in the general population. Indeed, the 3.1% prevalence figure for CTS in UK Biobank is close to the prevalence for clinically diagnosed CTS (3.8%) derived from one of the largest and most robust epidemiological studies of CTS prevalence^18^.

The second objective of this study was to compare BMI between CTS cases and controls in UK Biobank. There is broad consensus in the literature that obesity (as measured by raised BMI) is a risk factor for CTS. A meta-analysis of 58 such studies reported that obese individuals were more than twice as likely to develop CTS, with each one-unit increase in BMI increasing the risk of CTS by 7.4%^24^. Consistently, we found a sizeable BMI difference (>2 kg/m^2^) between CTS cases and controls in UK Biobank, and this effect was seen in both sexes. No mechanistic evidence currently exists to explain how raised BMI increases risk of CTS^5^, and it is not immediately clear if the effect might be mediated through co-morbidities of the obese such as diabetes. One hypothesis centres around obesity-related metabolic syndrome, which can lead to glycosylation of extracellular proteins and cause fat deposition in nerves, leading ultimately to nerve dysfunction^25^. Interestingly, our previous study into the genetic basis of CTS strongly implicated several genes and biological pathways pertaining to the extracellular matrix in the pathophysiology of CTS^12^.

The third aim of this study was to use the UK Biobank dataset to replicate previously reported associations between CTS and three disease phenotypes (diabetes, RA, and hypothyroidism), and to arrive at accurate estimates of CTS prevalence in individuals with these diseases.

In males and females combined, the odds ratio (OR) for developing CTS in diabetes, RA, and hypothyroidism were 2.31, 2.70, and 1.47, respectively. In all three diseases, matching on BMI as an additional variable in generating the case-control datasets attenuated this effect, with ORs falling to 1.75, 2.43, and 1.35. This finding is consistent with the notion that raised BMI associated with these three diseases is in part responsible for mediating the increased risk of CTS. Whereas diabetes in females conferred a greater risk for developing CTS than in males (OR 2.63 in females vs 1.99 in males), in RA and hypothyroidism, the opposite effect was seen (2.46 in females vs 3.75 in males for RA; 1.46 in females vs 1.61 in males for hypothyroidism). This may in part be an artefact of the relative over-representation of females in the RA and hypothyroidism case-control cohorts (67.6% and 83.2%, respectively), in conjunction with the fact that female CTS cases outnumber males by approximately 2:1 in UK Biobank.

Strong evidence exists for the co-existence of both type 1 and type 2 diabetes with CTS. A large meta-analysis by Pourmemari et al.^26^ found the pooled odds ratio for the association between CTS and diabetes was 1.97 (95% CI 1.56–2.49). Four of the studies in the meta-analysis had adjusted their estimates for BMI, and the pooled odds ratios in these four studies was 1.68 (95% CI 1.12–2.53), suggesting that diabetes is a risk factor independent of BMI. The odds ratios computed in our study (OR 2.31 – unadjusted; OR 1.75 – adjusted for BMI) are compatible with the confidence intervals reported in this meta-analysis.

RA is considered a relatively strong risk factor for CTS. Multivariate analysis in a large matched case-control study cohort (with >3,000 CTS cases) found that patients with RA are 2.23 times more likely to develop CTS^27^, and Karadag et al.^28^ reported evidence of CTS in 17% of females with RA, compared to 4% in age-matched controls. A 2016 meta-analysis of ten studies of 74,254 individuals reported a pooled OR of 2.88 (95% CI 2.25–3.69) for the association between RA and CTS^29^. The authors acknowledged that few of the primary papers adjusted their observed associations for BMI (a putative risk factor for RA), suggesting that the OR may have been overestimated. Consistent with this hypothesis, in our study, the OR for the association between CTS and RA of 2.70 fell to 2.43 when we adjusted for BMI. Notably, our OR estimates, as for DM, are very close to the figures derived from a previous meta-analysis.

There is reasonably strong evidence for a link between hypothyroidism and CTS, albeit less robust than for diabetes and RA. Although two studies have reported electrophysiologically diagnosed CTS in one-third of hypothyroid patients^30^^,^^31^, a 2014 meta-analysis only found a modest association between hypothyroidism and CTS in studies that controlled for potential confounders (OR 1.44, 95% CI 1.27–1.63)^32^. This figure accords closely with our estimated OR of 1.47 (1.35 on adjustment for BMI), adding to the evidence that there is a modest, but genuine, association between the two diseases.

Certain limitations of this study must be discussed. Firstly, all participants in UK Biobank are volunteers, meaning that the cohort is on the whole healthier than the population at large, due to sample selection bias (the so-called “healthy volunteer effect”)^33^. UK Biobank participants were found to be less likely to be obese and to smoke, and had fewer self-reported health conditions compared to nationally representative data sources^34^. The lower rates of obesity, in particular, is likely to result in an underestimation of CTS prevalence in UK Biobank. Secondly, as discussed earlier, diagnostic codes from hospital medical records in the form of ICD-10 and OPCS4 codes are only available from 1996 onwards; therefore, any diagnoses prior to this date are heavily reliant on participants’ self-report, introducing a potential source of recall bias. Thirdly, this study was restricted to participants of white British ancestry, motivated by the fact that these individuals constitute the majority of the UK Biobank cohort. As such, the relationships between CTS and various phenotypes found in this study cannot necessarily be extrapolated to individuals of other ethnicities, and it would be interesting for future studies to extend this work to include other ethnic groups.

Nevertheless, this study, to the best of our knowledge, represents the largest series of case-control studies to examine the epidemiological associations of CTS. By harnessing the statistical power of a population-based cohort of >400,000, we can provide strong evidence for several important phenotypic associations of CTS. The associations we found were remarkably similar to those previously published in the literature for all associated conditions. This robust reproducibility is important, and adds further weight to our conclusions.

CTS aetiology is complex and multi-factorial^35^, with increased adiposity likely to play a central role in pathogenesis. With the 2017 Health Survey for England reporting that 64% of adults in England are overweight or obese^36^, this finding has clear public health implications. To gain a more complete understanding of the relationships between these phenotypes with respect to CTS aetiology (and to disentangle cause-and-effect), future work in this field will need to consider genetic risk factors for CTS^12^ and use bioinformatic techniques such as Mendelian randomisation^37^. Medical research is increasingly driven by ‘big data’, and this study provides the proof-of-principle of the feasibility of using large population-based cohorts such as UK Biobank for epidemiological investigations into diseases that are important to plastic and hand surgeons.

## Conflict of Interest statement

None.

## Appendix Supplementary materials

Image, application 1
