# Supplementary material for: Replication of epidemiological associations of carpal tunnel syndrome in a UK population-based cohort of over 400,000 people
Source: J Plast Reconstr Aesthet Surg. 2022 Mar;75(3):1034–40. doi: 10.1016/j.bjps.2021.11.025 (PMC8982328; doi:10.1016/j.bjps.2021.11.025)
Supplement: Supplementary file 1 [file mmc1.docx]

**Supplementary Table 1. Diagnostic codes used to phenotype individuals with CTS, diabetes, rheumatoid arthritis and hypothyroidism.** All sub-codes of the ICD-10 codes shown below were included. Total case numbers for each disease are shown at the bottom of the table.

| CTS | Diabetes | Rheumatoid Arthritis | Hypothyroidism |
| --- | --- | --- | --- |
| ***ICD-10 codes*** | | | |
| G560 Carpal tunnel syndrome | E10 Insulin-dependent diabetes mellitus | M05 Seropositive rheumatoid arthritis | E02 Subclinical iodine-deficiency hypothyroidism |
|  | E11 Non-insulin-dependent diabetes mellitus | M06 Seronegative rheumatoid arthritis | E03 Other hypothyroidism |
|  | E13 Other specified diabetes mellitus |  |  |
|  | E14 Unspecified diabetes mellitus |  |  |
| ***Non-cancer illness code (self-reported)*** | | | |
| 1541: Carpal tunnel syndrome | 1220: Diabetes | 1464: Rheumatoid arthritis | 1226: Hypothyroidism |
|  | 1222: Type 1 Diabetes |  |  |
|  | 1223: Type 2 Diabetes |  |  |
| ***OPCS4 operative procedure codes*** | | | |
| A651 Carpal tunnel release | - | - | - |
| A692 Revision carpal tunnel release | - | - | - |
| ***Operation code (self-reported)*** | | | |
| 1501: Carpal tunnel surgery | - | - | - |
| **N = 12,312** | **N = 24,588** | **N = 6,711** | **N = 23,838** |
